# *N*-glycosylation of mouse TRAIL-R restrains TRAIL-induced apoptosis

**DOI:** 10.1038/s41419-018-0544-7

**Published:** 2018-05-02

**Authors:** Yann Estornes, Yves Dondelinger, Kathrin Weber, Inge Bruggeman, Adam Peall, Marion MacFarlane, Serge Lebecque, Peter Vandenabeele, Mathieu J. M. Bertrand

**Affiliations:** 1 VIB Center for Inflammation Research, Technologiepark 927, Zwijnaarde-Ghent, 9052 Belgium; 2 Department of Biomedical Molecular Biology, Ghent University, Technologiepark 927, Zwijnaarde-Ghent, 9052 Belgium; 3 Université Claude Bernard Lyon 1, INSERM 1052, CNRS 5286, Centre Léon Bérard, Centre de Recherche en Cancérologie de Lyon, Lyon, F-69373 France; 4 MRC Toxicology Unit, University of Leicester, Lancaster Road, Leicester, LE1 9HN UK; 5 Hospices Civils de Lyon, Centre Hospitalier Lyon-Sud, Service d’Anatomie Pathologique, 69495 Pierre Bénite Cedex, France

## Abstract

The sensitivity of cells to death receptor-induced apoptosis is commonly controlled by multiple checkpoints in order to limit induction of excessive or unnecessary death. Although cytotoxic in various cancer cells, tumor necrosis factor (TNF)-related apoptosis-inducing ligand (TRAIL) does not trigger apoptosis in most non-transformed cells. The molecular nature of the checkpoints that normally protect the cells from TRAIL-induced death are not fully understood. Endoplasmic reticulum (ER) stress has been reported to switch the sensitivity of human cells to the cytotoxic effect of TRAIL, suggesting that this cellular state perturbs some of these protective mechanisms. We found that tunicamycin (TU), but no other ER stress inducers, sensitized mouse fibroblasts and hippocampal neuronal cells to TRAIL-induced apoptosis. Importantly, the sensitization was specific to TRAIL and not caused by differences in ER stress induction. Instead, it relied on the inhibition of *N*-glycosylation of the mouse TRAIL receptor (mTRAIL-R). Inhibition of *N*-glycosylation did not alter cell surface expression of mTRAIL-R but enhanced its ability to bind TRAIL, and facilitated mTRAIL-R oligomerization, which resulted in enhanced death-inducing signaling complex (DISC) formation and caspase-8 activation. Remarkably, reconstitution of mTRAIL-R-deficient cells with a version of mTRAIL-R mutated for the three *N*-glycosylation sites identified in its ectodomain confirmed higher sensitivity to TRAIL-induced apoptosis. Together, our results demonstrate that inhibition of *N*-glycosylation of mTRAIL-R, and not ER stress induction, sensitizes mouse cells to TRAIL-induced apoptosis. We therefore reveal a new mechanism restraining TRAIL cytotoxicity in mouse cells.

## Introduction

Tumor necrosis factor (TNF)-related apoptosis-inducing ligand (TRAIL) is a member of the TNF family that has the ability to drive NF-κB-dependent expression of pro-inflammatory mediators and/or trigger apoptosis upon binding to its cognate receptors hTRAIL-R1 and hTRAIL-R2 (also known as DR4 and DR5, respectively) in human, or mTRAIL-R in mouse^1–5^. Apoptosis is not the default response of most cells to TRAIL, but occurs in various types of cancer cells^6,7^. This selective cytotoxicity has generated hope for the use of TRAIL as anti-cancer agent. Nevertheless, many tumor cells remain refractory to apoptosis induced by TRAIL, and ways to sensitize these transformed cells to the pro-apoptotic effect of this cytokine are currently under investigation^8–10^.

The reason why certain cancer cells spontaneously die upon TRAIL sensing is not fully understood, but indicates that these cells have lost some of the protective mechanisms that normally inhibit or restrain apoptosis induction. TRAIL-R-induced apoptosis relies on the binding of the adapter molecule Fas-associated death domain (FADD) to the intracellular portion of the receptor for the further recruitment of caspase-8. Within the generated death-inducing signaling complex (DISC), caspase-8 undergoes autocatalytic processing, which results in the release of its active form in the cytoplasm where it cleaves and activates the effector caspases-3 and -7, ultimately leading to the dismantlement of the cell^1,11^. This extrinsic TRAIL-R-induced apoptotic trigger can be amplified by engagement of the intrinsic apoptotic pathway via caspase-8-dependent activation of Bid, a proapoptotic Bcl-2 family member^12–14^. Low concentration of the translation inhibitor cycloheximide (CHX) is commonly used to switch the TRAIL response from survival to death in human- and mouse-resistant cells, indicating that TRAIL-induced apoptosis is normally actively repressed by proteins of short half-lives and/or translated in response to TRAIL sensing. The cellular expression of the caspase-8 homolog FLICE-inhibitory protein (cFLIP), which can be induced by NF-κB activation, appears to greatly influence the sensitivity of the cells to the proapoptotic effect of TRAIL^15,16^, similarly as for other death ligands of the TNF family. Indeed, cFLIP repression was shown to be sufficient to switch the TRAIL response to death in resistant cells of both normal and cancerous origin^17–19^. In addition to the translational control of cFLIP and other agonistic/antagonistic proteins, the sensitivity to TRAIL is also influenced by post-translational modifications. Among them, *O*-glycosylation of hTRAIL-R1 and hTRAIL-R2 is proposed to sensitize cancer cells to TRAIL-induced apoptosis by facilitating clustering of the receptors and, consequently, DISC assembly^20^.

Sensitization to TRAIL-induced apoptosis is also frequently reported in human cells undergoing endoplasmic reticulum (ER) stress, a cellular state of distress caused by accumulation of un- or misfolded proteins in the ER lumen. The unfolded-protein response (UPR), an integrated signaling network activated by eukaryotic cells in response to ER stress, initially aims at restoring protein homeostasis by reducing the protein load, but turns into a toxic signal triggering death when the cell is unable to cope with the severity of the stress^21^. Although the mechanism by which ER stress sensitizes human cells to TRAIL-induced apoptosis remains controversial, some studies explain it by the UPR-dependent downregulation of cFLIP and/or upregulation of hTRAIL-R1/2^22^^–^^28^. Apart from switching the sensitivity of human cells to the cytotoxic effect of TRAIL, the upregulation of hTRAIL-R2 by the UPR is also proposed to cause ligand-independent receptor clustering, DISC formation, and apoptosis induction in human cells undergoing unresolved ER stress^29–32^. In contrast with these later findings, we previously reported that mTRAIL-R is dispensable for ER stress-induced death in mouse embryonic fibroblast (MEFs)^33^, thereby questioning the general and/or conserved aspect of the ER stress-induced death mechanism described in human cells.

In this study, we found that tunicamycin (TU), but no other ER stress inducer, confers sensitivity of MEFs, mouse 3T3 fibroblasts, and HT-22 hippocampal neuronal cells to TRAIL-induced death. Importantly, we show that the sensitization is unrelated to ER stress induction, but instead originates from inhibition of *N*-glycosylation of mTRAIL-R. Our study therefore highlights differences in the sensitization of human and mouse cells to TRAIL-induced apoptosis in the context of ER stress, and reveals a new post-translational mechanism restraining TRAIL cytotoxicity in cells of mouse origin.

## Results

### Tunicamycin specifically sensitizes mouse cells to TRAIL-induced apoptosis

**Fig. 1:**
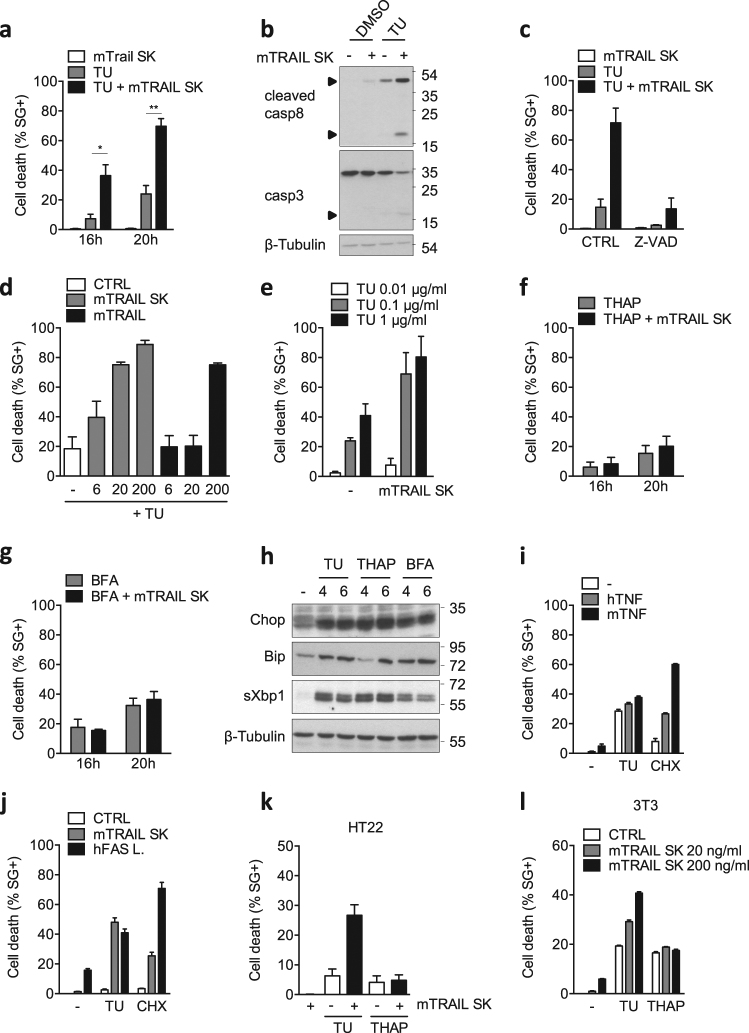
Tunicamycin specifically sensitizes mouse cells to TRAIL-induced apoptosis. **a** Cell death profiles of MEFs treated with 20 ng/ml recombinant mouse SuperKillerTRAIL (mTRAIL SK), 1 μg/ml tunicamycin (TU), or the combination of both. The percentage of cell death was measured over time using a Fluostar Omega fluorescence plate reader analyzing the SYTOX Green-positive cells. Error bars represent S.E.M. of three independent experiments. *P < 0.05; **P < 0.01**. b** MEFs were stimulated with TU in presence or absence of 20 ng/ml mTRAIL-SK for 15 h, and cell lysates were immunoblotted as indicated. Caspase cleavage products are indicated by arrowheads. Representative images of two independent experiments. **c** Cell death profile of MEFs pretreated with 20 μM Z-VAD-FMK (Z-VAD) or control (CTRL) followed by treatment with either mTRAIL-SK (20 ng/ml), TU, or TU + mTRAIL-SK for 18 h. Cell death was measured using a Fluostar Omega fluorescence plate reader. Error bars represent S.E.M. of two independent experiments. **d** Cell death profiles of MEFs stimulated with TU in combination or not with increasing doses (6, 20, and 200 ng/ml) of mTRAIL SK or mTRAIL for 20 h. Cell death was measured using a Fluostar Omega fluorescence plate reader. Error bars represent S.E.M. of two independent experiments. **e** Cell death profiles of MEF cells stimulated with 20 ng/ml mTRAIL-SK in combination or not with increasing doses of TU for 20 h. Cell death was measured using a Fluostar Omega fluorescence plate reader. Error bars represent S.E.M. of two independent experiments. **f, g** Cell death profiles of MEFs exposed to **f** 1 μM thapsigargin (THAP) or THAP + 20 ng/ml mTRAIL-SK; or **g** 0.5 μM brefeldin A (BFA) or BFA + 20 ng/ml mTRAIL-SK for 16 h and 20 h. Cell death was measured using a Fluostar Omega fluorescence plate reader. Error bars represent S.E.M. of three independent experiments. **h** MEFs were incubated with TU, THAP, or BFA for 4 and 6 h, and cell lysates were immunoblotted as indicated. Representative images of two independent experiments. **i** Cell death profiles of MEFs exposed to TU or 1.5 μg/ml translation inhibitor cycloheximide (CHX) in combination or not with 20 ng/ml of human (h) or mouse (m) TNF for 20 h. Cell death was measured using a Fluostar Omega fluorescence plate reader. Error bars represent S.E.M. of two independent experiments. **j** Cell death profiles of MEFs exposed to TU or 1.5 μg/ml CHX in combination or not with 20 ng/ml mTRAIL-SK or hFas Ligand (hFasL) for 16 h. Cell death was measured using a Fluostar Omega fluorescence plate reader. Results are representative of two independent experiments. Error bars represent S.E.M. of triplicates from a representative experiment. **k** Cell death profiles of HT22 cells treated for 24 h with 20 ng/ml mTRAIL-SK, or with TU or THAP in combination or not with mTRAIL-SK. Cell death was measured using a Fluostar Omega fluorescence plate reader. Error bars represent S.E.M. of two independent experiments. **l** Cell death profiles of 3T3 cells treated with mTRAIL-SK or with TU or THAP, in combination or not with mTRAIL-SK. Cell death was measured using an Incucyte ZOOM® system. Results are representative of two independent experiments. Error bars represent S.E.M. of duplicates from a representative experiment

Previous studies have reported that UPR-mediated upregulation of hTRAIL-R1 and hTRAIL-R2 can induce apoptosis in human cells by promoting hTRAIL-R2 clustering independently of TRAIL sensing, or by sensitizing the cells to the cytotoxic effect of TRAIL^22,24–30,32^. In contrast to these findings, we previously showed that mTRAIL-R is dispensable for ER stress-induced apoptosis in MEFs^33^. In order to test whether MEFs can still be sensitized to TRAIL under ER stress conditions, we stimulated the cells with TRAIL in the absence or presence of TU, a classical ER-stress inducer. Remarkably, while the MEFs were completely resistant to TRAIL used alone, its co-stimulation with TU greatly sensitized the cells to TRAIL-induced apoptosis, as monitored by plasma membrane permeabilization (Fig. 1a), caspase-8 and -3 processing (Fig.1b), and cell death inhibition in presence of the pan caspase inhibitor zVAD-fmk (Fig. 1c). The sensitization was dose dependent, both when using recombinant mouse SuperKillerTRAIL™ (mTRAIL-SK) or a less stable TRAIL ligand (mTRAIL) (Fig. 1d, e). Surprisingly, TRAIL-induced apoptosis was not observed when co-stimulating the cells with thapsigargin (THAP) (Fig. 1f) or brefeldin A (BFA) (Fig. 1g), despite their comparable potency at inducing the classical ER stress markers Chop, Bip, and the spliced form of Xbp1 (Fig. 1h). Importantly, TU treatment did not sensitize MEFs to TNF (Fig. 1i), indicating that TU did not confer general sensitization of mouse cells to death ligands of the TNF family. In line with previous studies^34–36^, we however found that TU also sensitized MEFs to Fas ligand (FasL) (Fig. 1j), which therefore served as a positive control in the experiment. Of note, CHX sensitized the cells to death in response to FasL, TNF, and TRAIL, but the sensitizing effect appeared stronger for FasL and TNF (Fig. 1i, j).

The specific sensitization to TRAIL-induced apoptosis by TU was not limited to MEFs but also observed in mouse HT-22 hippocampal neuronal cells (Fig. 1k) and 3T3 fibroblasts (Fig. 1l). This situation contrasted with the reported ER stress-dependent sensitization to TRAIL in human cells and that we confirmed in Hela and NCI-H292 cancer cells (Suppl. Figure 1a-b) as well as in nonmalignant nasopharyngeal NP69 cells (Suppl. Figure 1c). Taken together these results reveal that TU can sensitize mouse cells to TRAIL-induced apoptosis independently of ER-stress induction, and highlight differences between human and mouse TRAIL-R signaling that cannot be explained by the malignant state of the cells.

### TU induces appearance of a non-*N*-glycosylated form of mTRAIL-R

**Fig. 2:**
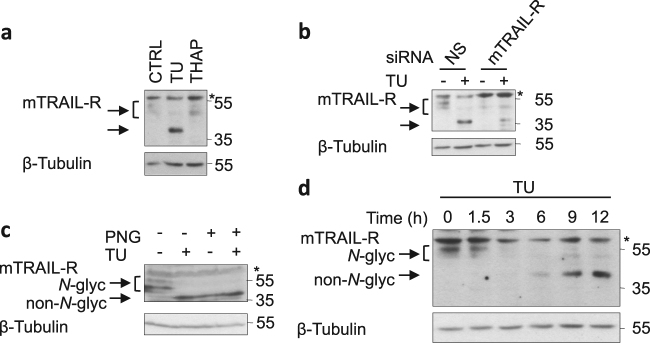
Tunicamycin induces appearance of a non-*N*-glycosylated form of mTRAIL-R. **a** MEFs were treated with CTRL, TU, or THAP for 17 h, and cell lysates were immunoblotted for mTRAIL-R and β-tubulin. Asterisk indicates non-specific signal. Representative images of three independent experiments. **b** MEFs were transfected with non-silencing (NS) or mTRAIL-R specific siRNA, and then treated with TU for 17 h. Cell lysates were immunblotted for mTRAIL-R and β-tubulin. Asterisk indicates non-specific signal. Representative images of three independent experiments. **c** Cell lysates of CTRL- or TU-treated MEFs were incubated at 37 °C for 1 h in presence or absence of PNGase F (PNG), and then immunoblotted for mTRAIL-R and β-tubulin. Asterisk indicates non-specific signal. Representative images of two independent experiments. **d** MEF cells were treated with TU for increasing times and cell lysates were immunblotted for mTRAIL-R and β-tubulin. Asterisk indicates non-specific signal. Representative images of three independent experiments

TU is an inhibitor of *N*-glycosylation, which let us hypothesize that the sensitizing effect of TU was originating from defective *N*-glycosylation. The fact that TU did not sensitize cells to TNF also suggested that it was affecting specific component(s) of the TRAIL signaling pathway. In line with this idea, we found that TU led to the appearance of a non-*N*-glycosylated form of mTRAIL-R. Indeed, TU treatment induced a mobility shift of mTRAIL-R that resulted in the detection of a sharp 40 kDa band instead of a diffuse signal around 50 kDa in control and THAP conditions (Fig. 2a). We confirmed that both signals were specific to mTRAIL-R by siRNA-mediated knockdown of the receptor (Fig. 2b) and demonstrated the non-*N*-glycolysated nature of the 40 kDa band by PNGase F treatment (an amidase that removes all *N*-glycans). Indeed, similar migration of mTRAIL-R was observed following TU treatment or digestion with PNGase F, and no further mobility shift was obtained when PNGase F was added to the TU-treated cell lysates (Fig. 2c). Full kinetics of TU stimulation indicated that the progressive loss of *N*-glycosylated mTRAIL-R was accompanied by the appearance of the de novo synthesized non-*N*-glycosylated receptor, which became predominant in the cell lysate starting around 6 h post stimulation (Fig. 2d).

### Non-*N*-glycosylated mTRAIL-R is expressed at the plasma membrane where it exhibits higher binding capacity for TRAIL

**Fig. 3:**
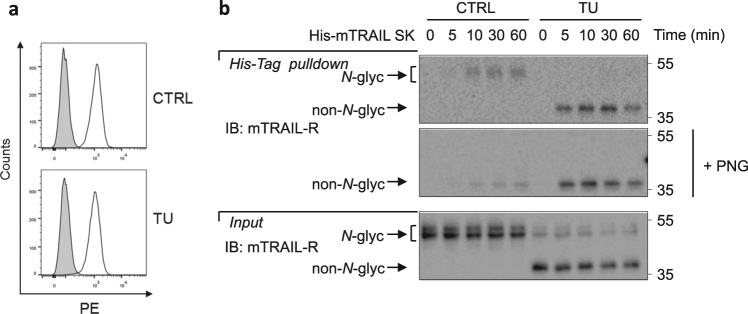
Non-*N*-glycosylated mTRAIL-R is expressed at the plasma membrane where it exhibits higher binding capacity for TRAIL. **a** Cell surface expression of mTRAIL-R in MEFs treated for 7 h with CTRL or TU, and analyzed by flow cytometry using a FACSVerse cytometer. Isotype-PE (filled histogram), mTRAIL-PE (open histogram). Representative histogram of three independent experiments. **b** Immunoblots under non-reducing conditions of mTRAIL-R of a His-Tag pulldown on MEFs pre-treated with TU or control for 7 h followed by mTRAIL-SK (500 ng/ml) treatment for increasing times. Before loading, pulldown samples were treated (+PNG) or not with PNGase F in non-reducing conditions. Representative images of at least two independent experiments

*N*-glycosylation supports the correct folding of proteins and is required for proteins to adopt their functional tertiary structures before reaching final destination^37,38^. In order to test whether the detected non-*N*-glycosylated mTRAIL-R is functional and expressed at the plasma membrane, we first compared cell surface expression of mTRAIL-R in control conditions and after 7 h of TU treatment, a timing at which cells predominantly expressed non-*N*-glycosylated mTRAIL-R (Figs. 2d, 3b). As shown in Fig. 3a, both *N*-glycosylated and non-*N*-glycosylated forms of the receptor were similarly expressed at the plasma membrane (Fig. 3a). Thus, non-*N*-glycosylated mTRAIL-R passes the quality control of protein folding in the ER and is properly addressed to the plasma membrane. The ability of the non-*N*-glycosylated receptor to sense TRAIL was then evaluated by performing a kinetic analysis of His-tag mTRAIL pulldowns in cells pre-stimulated, or not, with TU for 7 h. The fact that *N*-glycosylated mTRAIL-R appears as a diffuse signal around 50 kDa while non-*N*-glycosylated mTRAIL-R is detected as a sharp 40 kDa band renders difficult the comparison of the binding efficiencies of the two forms of the receptor (Suppl. Figure 2). To circumvent this problem, we treated the samples with PNGase F after the pulldown, and then analyzed them by immunoblot in non-reducing conditions. We indeed noticed that the anti-mTRAIL antibody used recognizes an epitope that is partially lost under reducing conditions (Suppl. Figure 2). By this methodological approach, we observed earlier and stronger binding of mTRAIL to the non-*N*-glycosylated form of mTRAIL-R, indicating a better affinity of non-*N*-glycosylated receptor for its ligand (Fig. 3b).

### Non-*N*-glycosylated mTRAIL-R triggers ligand-dependent apoptosis in TU-treated cells

**Fig. 4:**
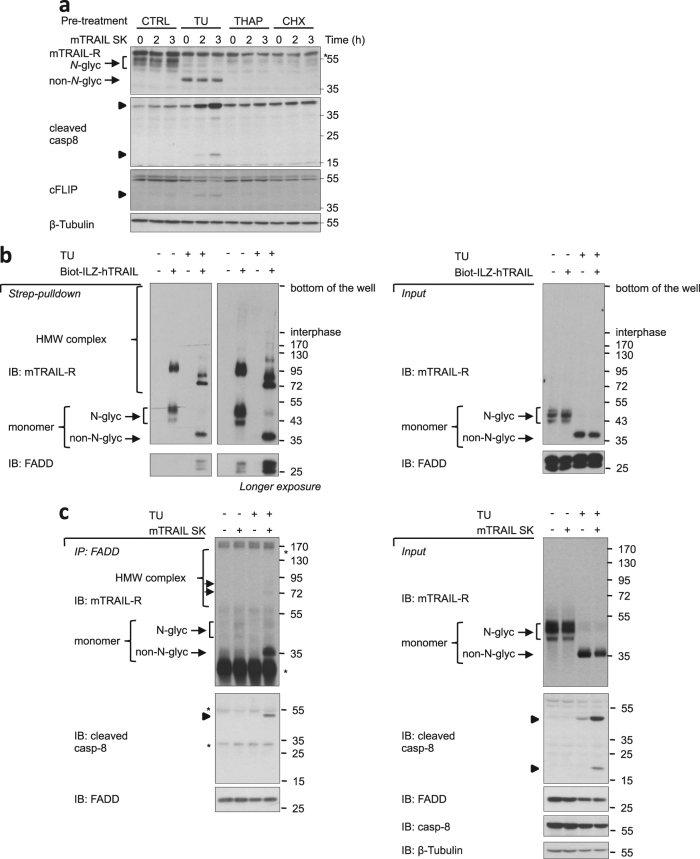
Non-*N*-glycosylated mTRAIL-R triggers ligand-dependent apoptosis in tunicamycin-treated cells. **a** MEFs were treated for 6 h with control or TU followed by stimulation with mTRAIL-SK (20 ng/ml) for the indicated times. Cell lysates were then immunoblotted as indicated. Caspase-8 and c-FLIP cleavage products are indicated by arrowheads. Asterisk indicates non-specific signal. Representative images of three independent experiments. **b** MEFs were exposed to TU or control for 7 h followed by Biotin-ILZ-hTRAIL (biot-ILZ-hTRAIL) (500 ng/ml) or CTRL treatment for 15 min. Cell lysates were subjected to streptavidin (Strep) pulldown and immunoblotted for mTRAIL-R and FADD under non-reducing conditions. Left panels: Strep pulldown. Right panels: Input. HMW high molecular weight. Representative images of two independent experiments. **c** MEFs were exposed to TU or control for 7 h followed by mTRAIL-SK (20 ng/ml) or CTRL treatment for 2 h. Cell lysates were subjected to FADD immunoprecipitation (IP:FADD) and immunoblotted as indicated. mTRAIL-R was detected under non-reducing conditions. Left panels: IP:FADD. Right panels: Input. Caspase-8 cleavage products are indicated by arrowheads. HMW high molecular weight. Asterisk indicates non-specific signal. Representative images of two independent experiments

The results obtained so far indicate that TU-dependent sensitization to TRAIL-induced apoptosis is associated with plasma membrane exposure of a non-*N*-glycosylated form of mTRAIL-R, but whether this modified receptor plays any role in TRAIL sensitization is currently unknown. To evaluate this possibility, we pretreated MEFs for 6 h with TU to induce full conversion of mTRAIL-R to its non-*N*-glycosylated state, and then tested the response of the cells to TRAIL stimulation. Remarkably, processing of caspase-8 was detected after only 2 h of mTRAIL-SK stimulation in the non-*N*-glycosylated mTRAIL-R-expressing cells (Fig. 4a). Such a sensitization was not observed in cells pretreated for 6 h with THAP or CHX (Fig. 4a), supporting the idea of increased cytotoxic potential of non-*N*-glycosylated mTRAIL-R.

Galectins bind *N*-acetyllactosamine present on complex-type *N*-glycans^39^, and Galectin-3 has been reported to negatively regulates TRAIL-induced death in human cancer cells, potentially by binding to hTRAILR1/R2 and altering their ligand-dependent internalization^40^. To test whether inhibition of mTRAIL-R *N*-glycosylation by TU releases an inhibitory role of galectins on mTRAIL-R, we stimulated the cells with kifunensine and swainsonine, respectively mannosidase I and II inhibitors that lead to the accumulation of proteins harboring high mannose-type *N*-glycans devoid of *N*-acetyllactosamine. In contrast to TU, neither kifunensine nor swainsonine sensitized MEFs to mTRAIL-SK-mediated cell death (Suppl. Figure 3a-b), indicating that the sensitization to TRAIL killing induced by inhibition of *N*-glycosylation of mTRAIL-R does not originate from impairment of galectins binding to mTRAIL-R *N*-glycans.

TRAIL-induced apoptosis relies on ligand-dependent receptor oligomerization, DISC formation, and auto-activation of caspase-8 within the DISC. We then analyzed DISC assembly by TRAIL pulldowns in MEFs pretreated or not with TU for 6 h (Fig. 4b, c). Interestingly, binding of TRAIL to the two versions of mTRAIL-R led to the detection, in non-reducing conditions, of high molecular weight (HMW) forms of the receptor that had different profiles. While *N*-glycosylated mTRAIL-R formed a single prominent oligomer at around 95 kDa, non-*N*-glycosylated mTRAIL-R was present in several oligomeric conformers with major bands detected around 80, 90, and 120 kDa (Fig. 4b). Non-*N*-glycosylated mTRAIL-R also recruited more FADD than its *N*-glycosylated counterpart, despite apparent similar efficiency in the amount of receptor pulled-down by the biotinylated Isoleucine Zipper (biot-ILZ) hTRAIL (Fig. 4b). Under similar conditions, FADD immunoprecipitation confirmed the increased association of FADD with non-*N*-glycosylated mTRAIL-R in response to mTRAIL-SK (Fig. 4c). Taken together these results indicate that in TU-treated MEFs, TRAIL-induced apoptosis is driven by a non-*N*-glycosylated form of mTRAIL-R that appears to have distinct oligomerization capacities and enhanced cytotoxic potential.

### mTRAIL-R is *N*-glycosylated on three asparagine residues in its ectodomain

**Fig. 5:**
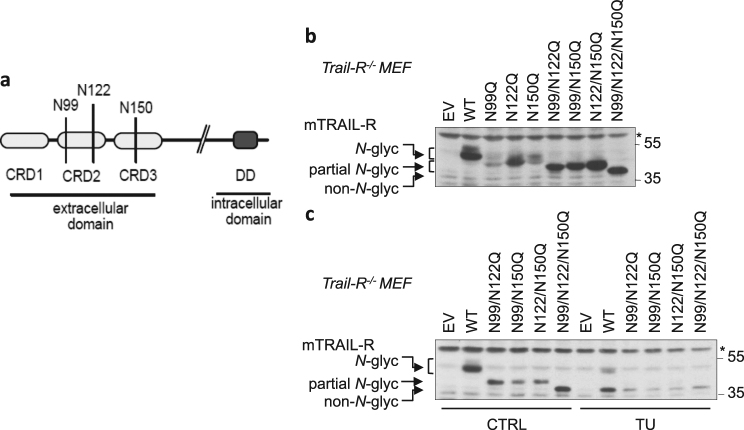
mTRAIL-R is *N*-glycosylated on three asparagine residues in its ectodomain. **a** Schematic presentation of the three putative *N*-glycosylation sites (NetGlyc 1.0 prediction) in the extracellular domain of mTRAIL-R. **b** *Trail-R*^*−*^^*/−*^ MEFs were transiently transfected with an empty vector (EV), wild-type (WT) mTRAIL-R, or *N*-glyc mTRAIL-R mutants pcDNA3 plasmids for 24 h, and cell lysates were immunoblotted for mTRAIL-R. Representative images of three independent experiments. **c** *Trail-R*^*−*^^*/−*^ MEFs were transiently transfected with an EV, WT mTRAIL-R, or *N*-glyc mTRAIL-R mutants pcDNA3 plasmids for 24 h in absence (CTRL) or presence of TU, and cell lysates were immunoblotted for mTRAIL-R. Representative images of three independent experiments

In silico analysis of the mTRAIL-R sequence with NetGlyc 1.0 predicted existence of three *N*-glycosylation sites on the ectodomain of the receptor, on Asparagine (N) residues N99, N122, and N150 (Fig. 5a). To test the presence of *N*-glycans on these potential acceptor residues, we mutated each of them separately and together, and then overexpressed the obtained mutants in *Trail-R*^*−/−*^ MEFs by transient transfection (Fig. 5b, c). We chose to perform Asparagine to Glutamine substitutions (N > Q) to maintain the polar character of the amino acid. Analysis of the migration profiles of the different mutants by reducing SDS PAGE revealed *N*-glycosylation of mTRAIL-R on each of the predicted sites (Fig. 5b). Importantly, only the triple N99/N122/N150Q mutant had a similar migration pattern as the non-*N*-glycosylated receptor generated by TU/PNGase F treatment (Figs. 5c, 2c), thereby demonstrating absence of additional *N*-glycosylation site in mTRAIL-R.

### *N*-glycosylation of mTRAIL-R restrains apoptosis induction

**Fig. 6:**
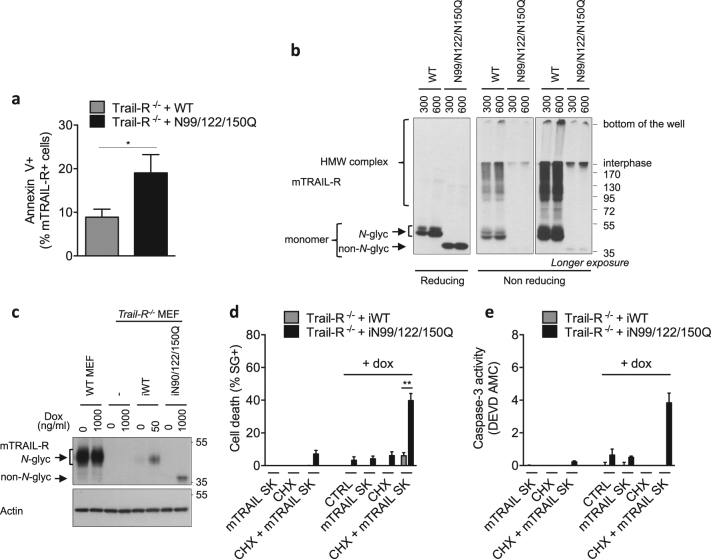
*N*-glycosylation of mTRAIL-R restrains apoptosis induction. **a** *Trail-R*^*−/−*^ MEFs were transiently transfected with 300 ng of pcDNA3 plasmids coding for a wild-type (WT) mTRAIL-R, or non-*N*-glyc mTRAIL-R N99/122/150Q mutant. After 24 h, the percentage of double positive mTRAIL-R+/Annexin V+ cells was analyzed by flow cytometry using a FACSVerse cytometer. Error bars represent S.E.M. of four independent experiments. *P < 0.05 in a ratio paired *t* test. **b** *Trail-R*^*−/−*^ MEFs were transiently transfected with 300 or 600 ng of pcDNA3 plasmids coding for WT or N99/122/150Q mutant mTRAIL-R. After 24 h, cell lysates were immunoblotted for mTRAIL-R in reducing vs non-reducing conditions. HMW high molecular weight. Representative images of at least two independent experiments. **c** *Trail-R*^*−/−*^ MEFs were stably transduced with viral particles coding for an inducible wild-type (iWT) mTRAIL-R, or non-*N*-glyc mTRAIL-R N99/122/150Q mutant (iN99/122/150Q). The cells were treated with doxycycline (Dox) at the indicated concentrations for 24 h, and cell lysates were immunoblotted for mTRAIL-R in non-reducing conditions and for actin in reducing conditions. Representative images of three independent experiments. **d, e** Cell death (**d**) and caspase-3 activity (**e**) profiles of *Trail-R*^*−/−*^ MEFs transduced as in **c**, and then treated with mTRAIL-SK (20 ng/ml), cycloheximide (CHX; 0.250 μg/ml), or the CHX/mTRAIL-SK combination for 24 h. Cell death and caspase-3 activity were measured using a Fluostar Omega fluorescence plate reader. Error bars represent S.E.M. of three (**d**) and two (**e**) independent experiments. **P < 0.01

Transient overexpression of death receptors often triggers ligand-independent death due to spontaneous aggregation of the receptors^2–4^. We observed that overexpression of N99/N122/N150Q mTRAIL-R in *Trail-R*^*−*^^*/−*^ MEFs resulted in increased ligand-independent apoptosis when compared to overexpression of WT mTRAIL-R (Fig. 6a and Suppl. Figure 4a). Of note, no sign of ER stress was detected in cells overexpressing WT or non-*N*-glycosylated mTRAIL-R (Suppl. Figure 4b). The difference in cell death induction was not originating from higher expression of the mutated receptor since cell surface staining showed similar percentages of cells expressing both forms of the receptor, and the mean intensity of the signal was even weaker for the N99/N122/N150Q mTRAIL-R-expressing cells (Suppl. Figure 4c-e). This was confirmed by comparing the expression of the two forms of mTRAIL-R by immunoblot in reducing conditions (Fig. 6b). However, analysis of the cell lysates in non-reducing conditions revealed clear differences in the oligomeric state of the receptor. WT mTRAIL-R oligomerized into several species of variable sizes, which resulted in the detection of a smear starting at around 90 kDa. In contrast, the N99/N122/N150Q mTRAIL-R mutant was mainly detectable at the interface between the stacking and the running part of the gel, or as a light signal corresponding to the monomeric form of the receptor, indicating a differential oligomerization potential into HMW complexes (Fig. 6b). To overcome the problem of spontaneous cell death induction, we next stably reconstituted the *Trail-R*^*−*^^*/−*^ MEFs with a Tet-on lentiviral system for doxycycline(dox)-inducible expression of WT (iWT) and iN99/N122/N150Q mTRAIL-R, which allowed lower expression level of mTRAIL-R (Fig. 6c). In order to reach similar expression level of WT vs N99/N122/N150Q mTRAIL-R, dox concentration was lowered from 1000 to 50 ng/ml for the iWT mTRAIL-R-reconstituted cells (Fig. 6c). Surprisingly, stimulation with mTRAIL-SK did not induce apoptosis, monitored by plasma membrane permeabilization and caspase-3 activity, in the iN99/N122/N150Q mTRAIL-R-expressing cells (Fig. 6d, e). This suggests that the sensitization caused by TU treatment in WT cells does not solely originate from the expression of the non-*N*-glycosylated mTRAIL-R. Alternatively, the absence of cell death induction could also result from the too low expression level of exogenous iN99/N122/N150Q mTRAIL-R. Because sensitization to TRAIL can be achieved by pretreatment with CHX^16^, we next evaluated the effect of inhibiting *N*-glycosylation of mTRAIL-R under these sensitizing conditions. Remarkably, mTRAIL-SK-induced apoptosis was much higher in the iN99/N122/N150Q mTRAIL-R-expressing cells (Fig. 6d, e), thereby further demonstrating the role of *N*-glycosylation of mTRAIL-R in restraining its cytotoxic potential. Together, our results demonstrate that *N*-glycosylation of mTRAIL-R in its ectodomain constitutes one of the mechanisms restraining the cytotoxic effect of TRAIL in murine cells.

## Discussion

Although cytotoxic in various human cancer cells, TRAIL does not trigger apoptosis in most non-transformed cells. Sensitization of resistant cells to TRAIL-induced apoptosis is generally obtained by co-stimulation with the translation inhibitor cycloheximide, and is believed to be caused, at least in part, by the inhibition of cFLIP translation^16^. The state of ER stress has also been reported to sensitize human cancer cells to the cytotoxic effect of TRAIL. Under these conditions, the sensitization is mainly reported to originate from upregulation of hTRAIL-R2^22–28^. We confirmed the ER stress-associated sensitization of human cells to TRAIL-induced apoptosis and showed that this effect was not specific to cancer cells. We found that resistant non-cancerous mouse cells can also be sensitized to TRAIL cytotoxicity when co-stimulated with the ER stress inducer TU. However, in clear contrast with the human situation, the sensitization was not caused by ER stress since neither thapsigargin nor brefeldin A reproduced the sensitization. The effect was also not the result of mTRAIL-R upregulation, but instead caused by expression of a non-*N*-glycosylated form of the receptor. We found that mTRAIL-R is *N*-glycosylated on three Asparagine resides (N99, N122, and N150) of its ectodomain, and demonstrated the protective effect of these post-translational modifications by reconstituting mTRAIL-R-deficient cells with the non-*N*-glycosylated mutated receptor. Since mTRAIL stimulation in MEFs did not lead to detectable activation of the NF-κB and MAPK pathways (Suppl. Figure 5a-b), it is reasonable to think that the sensitization to TRAIL killing induced by inhibition of *N*-glycosylation of mTRAIL-R does not originate from alteration of NF-κB or MAPK activation.

In human cells, TRAIL can trigger apoptosis by binding to hTRAIL-R1 and hTRAIL-R2. The sensitizing effect of TU to TRAIL-induced apoptosis has been attributed to the induced expression of hTRAIL-R2, but the implication of hTRAIL-R1 is less established. In contrast to hTRAIL-R2, hTRAIL-R1 also contains a *N*-glycosylation site in its ectodomain. Indeed, treatment of various human cancer cells with TU results in the detection of a faster migrating form of hTRAIL-R1, indicative of interference with the *N*-glycosylation pattern of hTRAIL-R1, but its upregulation is only reported in a limited number of sensitized cells^24,26,28,30,41^. It is therefore possible that inhibition of *N*-glycosylation by TU also generates a non-*N*-glycosylated hTRAIL-R1 with increased cytotoxic potential.

Surprisingly, a recent study reports that *N*-glycosylation of hTRAIL-R1 on N156 or of mTRAIL-R on N99/N122/N150 sensitizes rather than protects cells from TRAIL-induced apoptosis^42^. These results clearly contrast with our findings on mTRAIL-R, and the precise reason for the apparent discrepancy remains unclear. Differences between the cellular systems used in the two studies should, however, be pointed out. We demonstrated the effect of *N-*glycosylation of mTRAIL-R by reconstituting mTRAIL-R-deficient cells with the non-*N*-glycosylated mutant, thereby excluding any possible interference with endogenous mTRAIL-R. Importantly, our genetic results are also supported by the pharmacological inhibition of *N-*glycosylation by TU, whose sensitization to TRAIL-induced apoptosis is triggered by non-*N*-glycosylated mTRAIL-R.

Our results demonstrate a synergistic effect between TU and TRAIL, and we explain it molecularly by the greater killing potential of non-*N*-glycosylated mTRAIL-R. As a negative control, we used TNF to rule out the possibility that TU induces synergistic killing with all death receptor ligands (a situation observed for CHX). Fas/CD95 has previously been reported to be *N*-glycosylated^34,43^, and we found that TU also sensitized MEFs to FasL. However, in contrast to TRAIL, CHX massively sensitized the cells to death in response to FasL. These results may suggest that the effect of TU on FasL-mediated killing is mainly caused by alteration of prosurvival molecules expression. This hypothesis could explain the controversial reported role of *N*-glycosylation on the killing potential of Fas/CD95^34–36,43,44^.

The addition of *N*-glycans onto a nascent polypetide confers the protein with a polar character and also provides steric interference that influence inter- and intramolecular protein interactions to substantially modify the structure and function of proteins^37,38^. *N*-glycans assist the correct folding of newly synthesized proteins and is required for proteins to adopt their functional tertiary structures before reaching final destination^37,38^. We found that mTRAIL-R is *N*-glycosylated on three residues of its ectodomain. Surprisingly, inhibition of *N*-glycosylation of mTRAIL-R did not affect its exposure on the plasma membrane but increased its ability to associate with TRAIL. Further studies will be required to directly compare these results with the reported non-altered binding of TRAIL to non-*N-*glycosylated hTRAIL-R1^42^. In addition, non-*N*-glycosylated mTRAIL-R showed differential oligomerization potential associated with facilitated DISC assembly. Analysis of the receptor by non-reducing SDS PAGE revealed formation of SDS-detergent resistant, HMW complexes stabilized by disulfide bonds. Since the *N*-glycosylation sites of mTRAIL-R reside in proximity to Cysteine residues, it is conceivable that the addition of *N*-glycans sterically interferes with the formation of disulfide bonds, thereby altering receptor oligomerization and DISC assembly.

Our results suggest that TU-induced sensitization of MEFs to TRAIL-mediated apoptosis is not solely originating from plasma membrane expression of non*-N*-glycosylated mTRAIL-R. Indeed, TRAIL exposure was not sufficient to induce apoptosis in mTRAIL-R-deficient MEFs reconstituted with the non*-N*-glycosylated receptor. Alternatively, this could be explained by the low level of receptor expression in the reconstituted cells. Nevertheless, the protective role played by *N*-glycosylation was demonstrated when TRAIL was combined to low levels of cycloheximide. Indeed, under these sensitizing conditions, apoptosis induction was much higher in the non*-N*-glycosylated mTRAIL-R-expressing cells. These results indicate that *N*-glycosylation of mTRAIL-R is probably not the most limiting checkpoint in the apoptotic pathway but serves to fine-tune the sensitivity of the cells. Further work will therefore be needed to identify the pathophysiological conditions that affect *N*-glycosylation of the receptor.

## Materials and methods

### Cell cultures

Wild-type (WT) MEFs were isolated from E12.5 littermate embryos and immortalized with SV40 large T antigen, as previously described^45^. Trail-R^−/−^ MEFs were a kind gift of Dr. Mak (UHN, Toronto, Canada). MEF, immortalized mouse hippocampal HT22, mouse fibroblasts 3T3, and human cervix cancer HeLa cells were cultured in high glucose Dulbecco’s modified Eagle’s medium (DMEM) supplemented with 10% fetal calf serum and l-glutamine (200 mM). Human lung cancer NCI-H292 were grown in RPMI 1640 medium supplemented with 10% fetal calf serum, HEPES, NaPy, and l-glutamine (200 mM). Immortalized nonmalignant human nasopharyngeal epithelial NP69 cells were grown in KSFM supplemented with 10% FCS. The sequence encoding WT mTRAIL-R was obtained from the pcR3-mTRAIL-R plasmid, a kind gift of Dr. Micheau (University of Bourgogne Franche-Comté, Dijon, France). The sequence was cloned into pENTR3C using the CloneEZ PCR Cloning Kit (GenScript, Piscataway, NJ, USA), and substitution of the Asparagine residues into Glutamine residues was achieved by QuikChange® Site-Directed Mutagenesis Kit (Agilent, Santa Clara, US). Sequences were then transferred into pcDNA3, and custom-made doxycyline-inducible pDG2(blast)-rtTA3-FLAG lentiviral destination vectors^46^ using the LR Gateway recombination system (Life Technologies, Carlsbad, CA, USA). Transient overexpression of mTRAIL-R mutants in *Trail-R*^−*/*−^ MEFs was done by transfection of pcDNA3 plasmids using JetPRIME® transfection reagent (Polyplus-transfection® SA, Illkirch, France), following the manufacturer’s recommendations. mTRAIL-R reconstitutions were also achieved by lentiviral transduction. Viral particles were produced by transfecting HEK293T cells using calcium phosphate with a pDG2(blast)-rtTA3-FLAG vector containing the different forms of mTRAIL-R, together with the lentiviral viral packaging vectors pMD2-VSVG and pCMV-∆R8.9.1. The medium was changed after 6 h, and the virus-containing supernatant was collected after 24 and 48 h and used to transduce the *Trail-R*^−*/*−^ MEFs. Stable reconstituted *Trail-R*^*−*^^*/*−^ MEFs were positively selected for 2 days by the addition of blasticidin (10 µg/ml) in the cell culture medium.

### Reagents and antibodies

Recombinant mouse SuperKillerTRAIL™ (mTRAIL-SK) (ALX-201-130-C020), mTRAIL (BML-SE722-0100), and human TRAIL-SK (ALX-201-115) were purchased from Enzo Life Sciences GmbH (Lörrach, Germany). Tunicamycin (#T7765), thapsigargin (#T9033), and brefeldin A (#B7651) were obtained from Sigma-Aldrich (St. Louis, MO, USA), and used at 1 µg/ml, 1 µM, and 0.5 µM, respectively, unless stated otherwise. Kifunensine (#K1140) and swainsonine (#S9263) were from Sigma. Z-VAD-fmk (Bachem, Bubendorf, Switzerland; #N-1510) was used at 20 µM. Recombinant human TNF was produced in our laboratory, purified to at least 99% homogeneity, had a specific biological activity of 3–107 IU/mg, and was used at 600 IU/ml (20 ng/ml). Cycloheximide was from Sigma-Aldrich (#C7698). PNGase F was a kind gift of Prof. Dr. Nico Callewaert (VIB-UGent, Center of Medical Biotechnology, Gent, Belgium). Biotin-IsoLeucine Zipper (Biot-ILZ)-hTRAIL was produced in the lab of Prof. MacFarlane (MRC Toxicology Unit, Leicester, United Kingdom). Antibodies were purchased from the following companies: anti-spliced Xbp-1 (BioLegend, San Diego, CA, USA; #619501), anti-CHOP (Cell Signaling, Danvers, MA, USA; #2895S), anti-Bip (BDTransduction Laboratories, San Jose, CA, USA; #610978), anti-caspase-8 (Abnova, Jhongli, Taiwan; #MAB3429), anti-cleaved caspase-8 (Cell Signaling; #9429), anti-caspase-3 (Cell Signaling; #9662), anti-β-tubulin (Abcam, Cambridge, UK; #ab6046-200), anti-cFLIP (Adipogen, San Diego, CA, USA; #AG-20B-0005), anti-mTRAIL-R (Santa Cruz Biotechnology, Dallas, TX, USA; #sc-57086), anti-FADD (Enzo #ADI-AAM-212), phospho-p65 (Cell Signaling; #3033), p65 (Cell Signaling; #8242), phospho-ERK (Cell Signaling; #9101), ERK (Cell Signaling; #4696), phospho-IκB (Cell Signaling; #9246), IκB (Cell Signaling; #7814, and Santa Cruz Biotechnology, #sc-371), phospho-MK2 (Cell Signaling, #3007), and MK2 (Cell Signaling, #3042). Unless stated otherwise in the figure legends, all immunoblots were performed in reducing conditions.

### Analysis of cell death and caspase-3 activity

Cell death and caspase-3-like activity were analyzed using the Fluostar Omega fluorescent plate reader (BMG Labtech GmbH, Ortenberg, Germany) as previously described^47^. Briefly, the cells were seeded in 96-well plates and were then treated with the indicated compounds in the presence of 5 µM SYTOX Green (SG) (Life Technologies) and in absence or presence of 20 µM DEVD-AMC (PeptaNova GmbH, Sandhausen, Germany). The maximal SG fluorescence was achieved by full permeabilization of the cells using 0.05% Triton X-100, and the percentage of cell death was calculated as (induced SG fluorescence−background SG fluorescence)/(maximal SG fluorescence−background SG fluorescence)×100. Cell death was also analyzed with an Incucyte ZOOM® system (Essen BioScience, Michigan, USA) analyzing SG positive cells. The 100% SG-positive cells were achieved by permeabilization of the cells using 0.02% Triton X-100. For some experiments, death of mTRAIL-R-positive cells was analyzed by flow cytometry. In this case, cells were resuspended in Annexin V-binding buffer containing Annexin V-APC (BD Biosciences, San Jose, CA, USA) and SYTOX Blue (Life Technologies) shortly before analysis by flow cytometry (FACSVerse, Becton Dickinson, Franklin Lakes, NJ, USA). The data were then analyzed using FlowJo software. Expression of the different inducible forms of mTRAIL-R was induced 24 h prior to cell death analysis by addition of doxycycline at the concentrations indicated in the figures.

### PNGase F and EndoH digestions

Cells were lysed in Laemmli’s buffer and the proteins were precipitated by adding two volumes of ice-cold 100% acetone for 30 min at 4 °C. Samples were then centrifugated at 13,000 × *g* for 5 min and the supernatant was discarded. Pellets were resuspended in water and denaturated using Glycoprotein Denaturing Buffer (New England BioLabs, Ipswich, MA, USA). PNGase F or EndoH (#P0702, New England BioLabs) was added or not, and the samples were incubated at 37 °C for 1 h. For some experiments, rapid PNGase F non-reducing format (#P0711, New England BioLabs) was used. Briefly, proteins were precipitated as above and pellets were resuspended in water-containing Rapid PNGase F (non-reducing format) buffer, and then incubated 5 min at 75 °C. Rapid PNGase F (non-reducing format) was added, and the samples incubated at 50 °C for 10 min. Laemmli’s buffer was added and the samples were boiled before analysis by immunoblots.

### Cell surface expression of mTRAIL-R

Plasma membrane expression of mTRAIL-R was achieved by flow cytometry. Cells were harvested and resuspended in cold PBS containing 0.5% FCS. The cells were then incubated in PBS-0.5% FCS containing anti-mTRAIL-R-PE (eBioscience, San Diego, CA, USA; # 12-5883) or isotype control at 4 °C for 30 min. Cells were then washed three times in cold PBS-0.5% FCS before analysis by the cytometer (FACSVerse). The data were then analyzed using FlowJo software.

### FADD immunoprecipitation

Following stimulation, cells were washed with cold PBS and then lysed in cold lysis buffer (10 mM Tris-HCl (pH7.5), 150 mM NaCl, 1% NP-40, and 10% glycerol), supplemented with EDTA-free protease inhibitor cocktail tablets (Roche Diagnostics, Basel, Switzerland) and phosphatase inhibitor cocktail tablets (Roche Diagnostics). Endogenous FADD was immunoprecipitated from the cleared lysates overnight at 4 °C using anti-FADD antibody (Santa Cruz Biotechnology, Dallas, TX, USA; #sc-6036) coupled to G beads. The beads were then recovered by centrifugation, and immunoprecipitates were washed three times in cold lysis buffer before elution in Laemmli’s buffer. Immunoprecipitates were then analyzed by immunoblots performed in reducing condition unless stated otherwise in the figure legends.

### mTRAIL-R pulldown assay

Strep-pulldown assay was performed as previously described^48^. Briefly, cells were seeded in 150 mm dishes, and treated or not the day after with TU for 7 h. The cells were then pre-cooled at 4 °C before adding Biot-ILZ hTRAIL at 500 ng/ml for 45 min at 4 °C to facilitate “loading” of TRAIL-R. The cells were switched to 37 °C for 15 min and then immediately washed with cold PBS and lysed in cold lysis buffer (30 mM Tris/HCl (pH 7.5), 150 mM NaCl, 10% glycerol, 1% Triton X-100) supplemented with EDTA-free protease inhibitors (Roche Diagnostics). Biot-ILZ hTRAIL-bound complexes were precipitated using streptavidin magnetic beads (Dynabeads^TM^ M-280 Streptavidin, Thermo Fisher Scientific, Waltham, MA USA) overnight at 4 °C. Beads were then washed three times with cold lysis buffer, and complexes eluted in Laemmli’s buffer before analysis by immunoblot. For His-Tag pulldown, cells were seeded and treated with TU as above. Cells were harvested, resuspended in 1 ml medium, and let recover for 30 min. Cells were then treated for increasing times with 500 ng/ml mTRAIL SK at 37 °C, washed with cold PBS, and lysed as above. mTRAIL SK-bound complexes were precipitated using His-Tag Dynabeads^TM^ magnetic beads (Thermo Fisher Scientific) overnight at 4 °C, and complexes eluted in Laemmli’s buffer before analysis by immunoblots performed in reducing condition unless stated otherwise in the figure legends.

### Statistical analysis

Unless stated otherwise, two-tailed unpaired *t*-test was used for statistical analysis. *P < 0.05 and **P < 0.01 were considered as significant.

## Electronic supplementary material

Supplemental Figures
