# Supplementary material for: N-glycosylation of mouse TRAIL-R restrains TRAIL-induced apoptosis
Source: Cell Death Dis. 2018 May 2;9(5):494. doi: 10.1038/s41419-018-0544-7 (PMC5931557; doi:10.1038/s41419-018-0544-7)

Supplemental Figure legends

Supplemental Figure 1. **Effect of tunicamycin *versus* thapsigargin on TRAIL-induced death in malignant and nonmalignant human cell lines.** (**a-b**) Cell death profiles of (**a**) HeLa (cervix) and (**b**) NCI-H292 (lung) cancer cells treated with hTRAIL-SK (20 ng/ml), or with TU (1µg/ml) or THAP (0.5µM) in combination or not with hTRAIL-SK. Cell death was measured using a Fluostar Omega fluorescence plate reader analyzing the SYTOXGreen positive cells. Results are representative of two independent experiments. Error bars represent S.E.M. of triplicates from a representative experiment. (**c**) Cell death profiles of nonmalignant NP69 (nasopharyngeal) epithelial cells treated with hTRAIL-SK (20 ng/ml) or with (1µg/ml) or THAP (0.5µM) in combination or not with hTRAIL-SK. Cell death was measured using an Incucyte ZOOM® system analyzing the SYTOXGreen positive cells. Error bars represent S.E.M. of two independent experiments.

Supplemental Figure 2. ***N*-glycosylated and non-*N*-glycosylated mTRAIL-R bind TRAIL.** Immunoblots under non-reducing (upper panels) and reducing (lower panels) conditions of mTRAIL-R of a His-Tag pulldown on MEFs pre-treated with TU or control for 7h followed by mTRAIL-SK (500 ng/ml) treatment for increasing times. Representative images of three independent experiments.

Supplemental Figure 3. Mannosidase I and II inhibitors do not sensitize MEFs to TRAIL-induced death. (**a-b**) Cell death profiles of MEFs stimulated with 20 ng/ml mTRAIL-SK in combination or not with (**a**) mannosidase I inhibitor kifunensine or (**b**) mannosidase II inhibitor swainsonine for the indicated times. Cell death was measured using a Fluostar Omega fluorescence plate reader. Error bars represent S.E.M. of two independent experiments. (**c**) MEFs were treated with increasing concentrations of swainsonine or kifunensine for 24h, and cell lysates were immunoblotted as indicated. Lysates from TU-treated cells for 7h are used as control for non-*N*-glyc mTRAIL-R mobility shift. (**d**) Cell lysates of MEFs treated with increasing doses of kifunensine were incubated at 37°C for 1h in presence or absence of PNGase F (PNG) or endoglycosidase H (EndoH), and then immunoblotted as indicated.

The efficacy of kifunensine and swainsonine to induce accumulation of mTRAIL-R harboring high mannose-type N-glycans is validated by a mobility shift of mTRAIL-R on SDS PAGE upon kifunensine treatment (suppl. Fig. 3c), and by the acquired sensitivity of mTRAIL-R *N*-glycans to EndoH digestion upon swainsonine treatment (suppl. Fig 3d).

Supplemental Figure 4. **Ectopic expression of wild-type mTRAIL-R *vs* non-*N*-glyc mTRAIL-R N99/122/150Q mutant in *Trail-R$^{-/-}$* MEFs.** (**a**) *Trail-R-/-* MEFs were transiently transfected with pcDNA3 plasmids coding for a wild-type (WT) mTRAIL-R, or non-*N*-glyc N99/122/150Q mTRAIL-R mutant (N99/122/150Q) in presence or absence of Z-VAD (20μM) . After 24h, cell lysates were immunoblotted as indicated. Caspase cleavage products are indicated by arrowheads. Representative images from at least two independent experiments. (**b**) *Trail-R$^{-/-}$* MEFs were transfected as in (**a**) and cell lysates were immunoblotted as indicated. MEFs treated or not with TU for 7h serve as control. Representative images from at least four independent experiments. (**c**) *Trail-R$^{-/-}$* MEFs were transfected as in (**a**), and after 24h the percentage of plasma membrane mTRAIL-R positive cells was measured by flow cytometry using a FACVerse cytometer. Representative contour plots of four independent experiments. (**d**) Quantification of the percentage of mTRAIL-R positive cells shown in **(c)**. Error bars represent S.E.M. of five independent experiments. ns, not significant. (**e**) Mean Fluorescence intensity (MFI) of the mTRAIL-R staining shown in (**c**). Error bars represent S.E.M. of five independent experiments. *$P<0.05$.

Supplemental Figure 5. No effect of mTRAIL-SK on the activation levels of p65, ERK, and MK2 in MEFs. (**a**) MEFs were treated for 7h with control or TU (1μg/ml) and then followed by stimulation with mTRAIL-SK (20 ng/ml) for the indicated times. Cell lysates were then immunoblotted as indicated. Lysates from MEFs treated with TNF (20 ng/ml) for the indicated times serve as controls. (**b**) *Trail-R-/-* MEFs stably expressing an inducible wild-type (iWT) mTRAIL-R, or non-N-glyc mTRAIL-R N99/122/150Q mutant (iN99/122/150Q) were treated for 24h with 1000 or 50 ng/ml doxycycline (Dox), respectively. The cells were then stimulated with mTRAIL-SK (20 ng/ml) for the indicated times, and cell lysates were immunoblotted as indicated.

Supplemental Figure 1

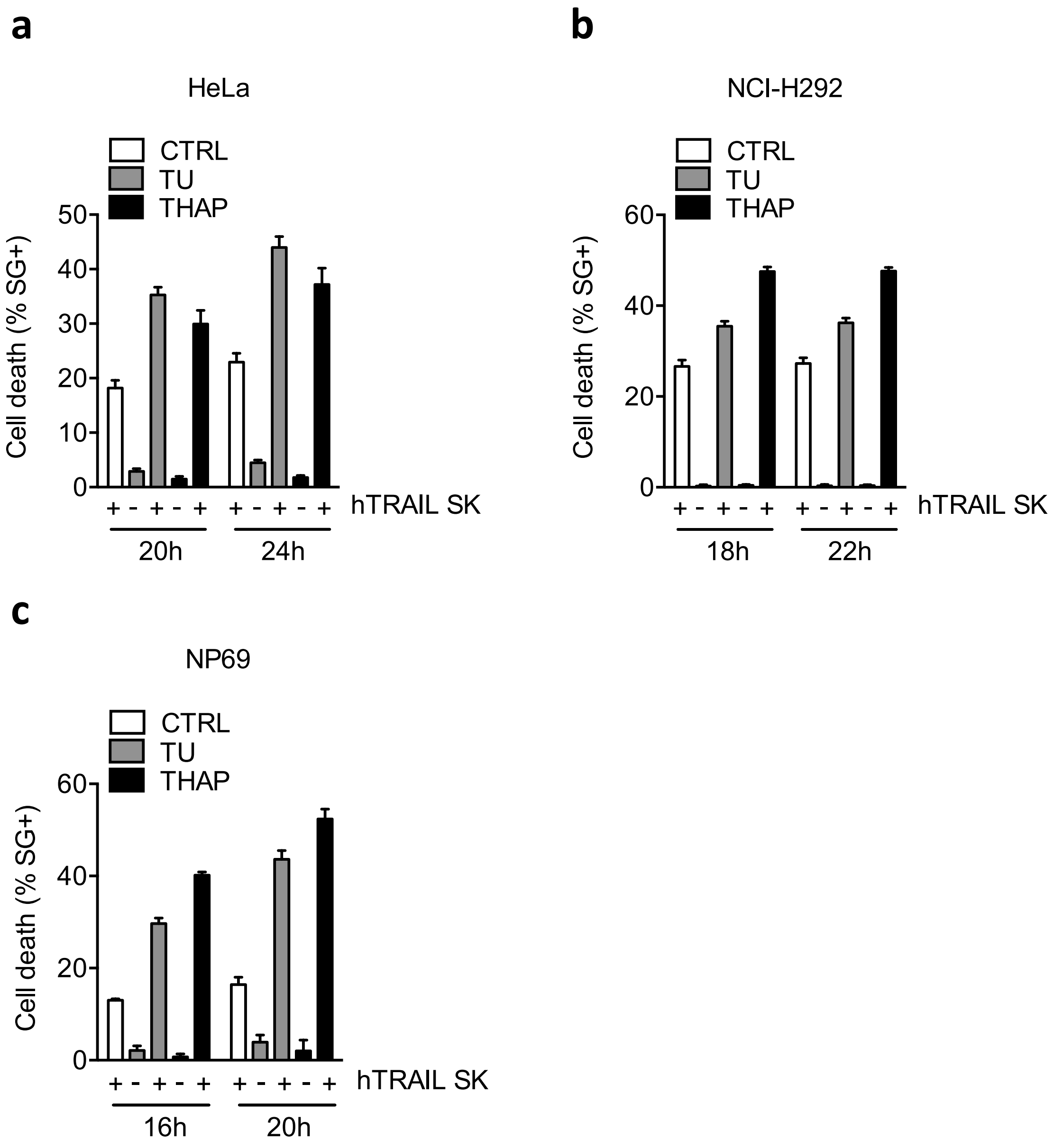

Supplemental Figure 2

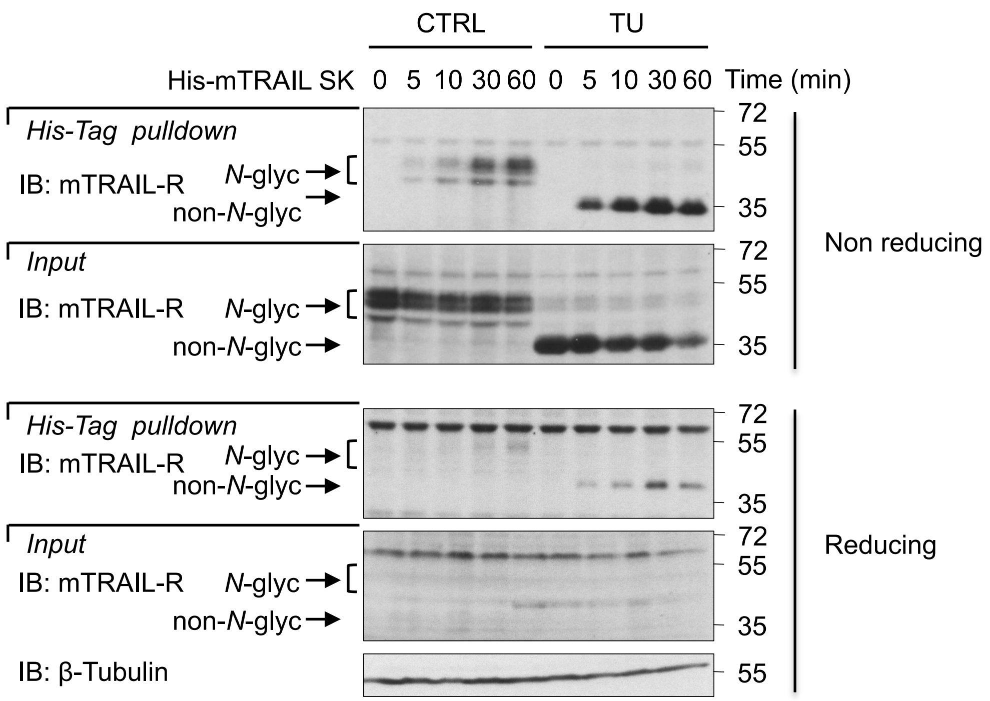

Supplemental Figure 3

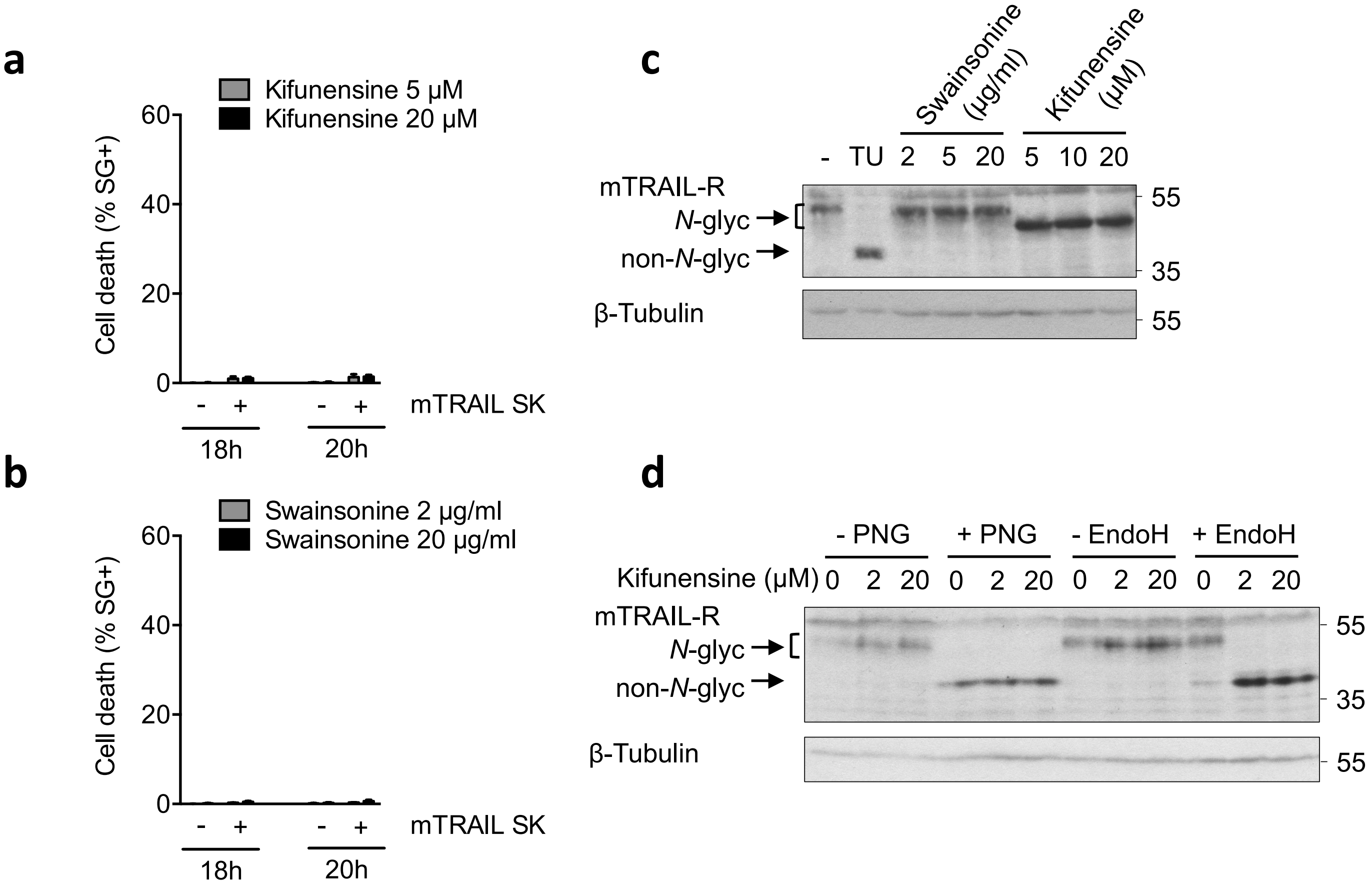

Supplemental Figure 4

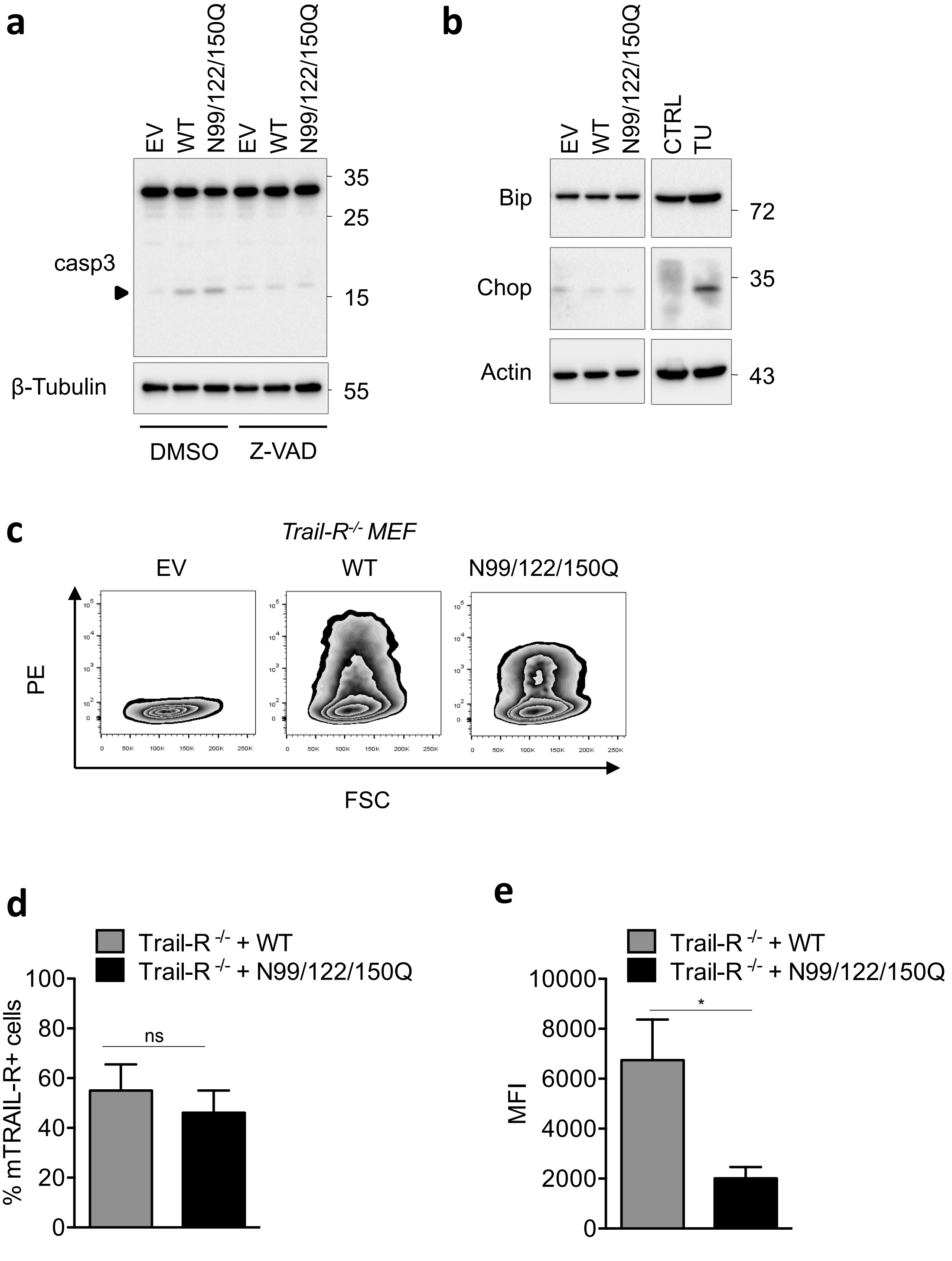

Supplemental Figure 5

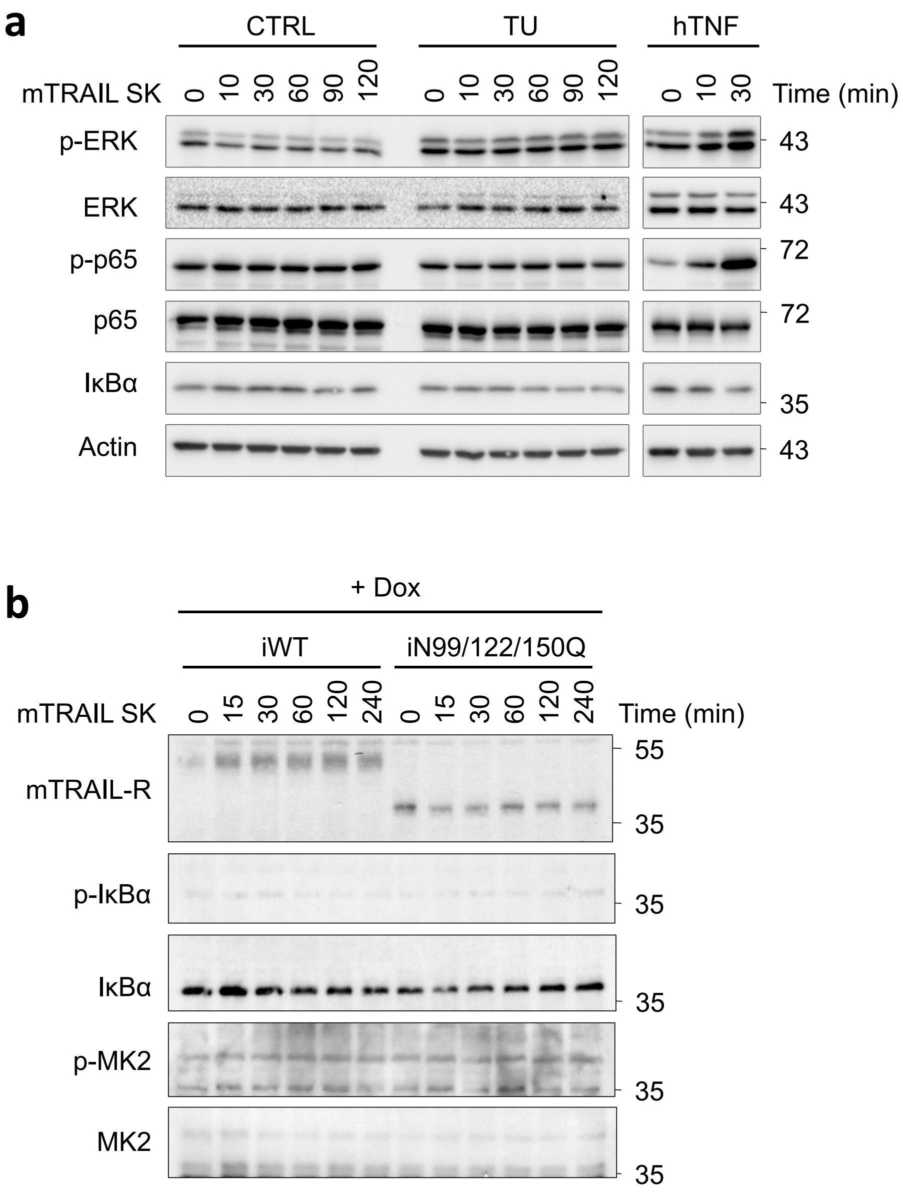

Supplement: Supplementary file 1 — Supplemental Figures [file 41419_2018_544_MOESM1_ESM.pdf]
